# Evaluating the impact of social support services on tuberculosis treatment default in Ukraine

**DOI:** 10.1371/journal.pone.0199513

**Published:** 2018-08-09

**Authors:** Martha Priedeman Skiles, Siân L. Curtis, Gustavo Angeles, Stephanie Mullen, Tatyana Senik

**Affiliations:** 1 MEASURE Evaluation Project, Carolina Population Center, University of North Carolina at Chapel Hill, Chapel Hill, North Carolina, United States of America; 2 Department Maternal and Child Health, Gillings School of Global Public Health, University of North Carolina at Chapel Hill, Chapel Hill, North Carolina, United States of America; 3 John Snow Inc., Rosslyn, Virginia, United States of America; 4 International Research Agency IFAK Institut, Kyiv, Ukraine; Agencia de Salut Publica de Barcelona, SPAIN

## Abstract

Ukraine is among the top 20 highest drug-resistant tuberculosis burden countries in the world. Driving the high drug-resistant tuberculosis rates is an unchecked treatment default rate. This evaluation measures the effect of social support provided to tuberculosis patients at risk of defaulting on treatment during outpatient treatment. Five tuberculosis patient cohorts, served in three oblasts from 2011 and 2012, were constructed from medical records to compare risk factors for default, receipt of social services, and treatment outcome. Regression analyses were used to identify risk factors predictive of treatment default and to estimate the impact of the social support program on treatment default, controlling for risk, disease status, and demographics. In 2012, tuberculosis patients receiving social support in Ukraine reduced their probability of defaulting on continuation treatment by 10 percentage points compared to high-risk patients who did not receive social support in 2012 or 2011. Treatment success rates for the high-risk patients receiving social support were comparable to the low-risk cohorts and significantly improved over the high-risk comparison cohorts. Further research is recommended to quantify the costs and benefits for scaling-up social support services, evaluate social support program fidelity, identify which populations respond best to select services, and what barriers might still exist to achieve better adherence. With that information, tailoring programs to most effectively reach and serve clients in a patient-centered approach may reap substantial rewards for Ukraine.

## Introduction

Tuberculosis (TB) remains a global threat with an estimated 1.8 million deaths in 2015; approximately 14 percent attributed to multi-drug resistant (MDR-TB) and rifampicin resistant (RR-TB) tuberculosis [1]. Advances in case detection and treatment regimens have dramatically reduced incidence and mortality [2,3]; yet lengthy and complex treatment regimens continue to take a toll on treatment completion and success rates. Patients who default on TB treatment continue to contribute to the infectious disease burden of the community [4], increase their risk of developing MDR-TB [5–6], and are at an increased risk for TB-related mortality [7].

According to the WHO 2016 report, Ukraine is among the top 20 highest drug-resistant TB burden countries in the world. National survey data estimate 25% of new incident cases and 58% of previously treated cases are MDR/RR-TB cases, resulting in approximately 22,000 cases per year. Driving these increasing MDR/RR-TB rates is an unchecked treatment default rate. Among the 2014 national TB cohort, treatment success was only 72% compared to 83% globally [1].

Patient predictors for treatment default vary across Europe and Central Asia. In Spain, Cayla and colleagues found that immigrants, patients living alone, patients previously treated, and injection drug users (IDU) were at higher risk for default [8]; whereas in Russia, the homeless, unemployed and alcoholics were at highest risk [9]; in Moldova patients who were homeless, living alone, less educated, or living for extended periods outside the country were at risk [10]; and in Estonia alcohol abuse, unemployment, MDR-TB, urban residence and previous incarceration increased risk [11]. Timing of default was found to be heterogeneous across a large meta-analysis by Kruk and colleagues [6]. In Uzbekistan, the first two-month intensive phase led to high rates of default, in Moldova the risky period was between intensive and continuation treatment, while data overall suggest that lengthy treatment during the continuation phase is the most likely period for defaulting [6].

Strategies to improve treatment adherence and success center on directly observed therapy short-course (DOTS) with adaptations to different clinical service and social environments. DOTS has been implemented in clinical settings, through home visits by medical personnel, use of community volunteers, and DOTS by family members [12]. Patient incentives are sometimes included to improve adherence, most commonly periodic food packages, transportation vouchers, and cash payments. However, evaluation of the impact of these strategies on treatment success remains inconclusive [13–14,2,12].

The standard TB treatment for smear-positive patients in Ukraine covers 2–4 months of intensive inpatient therapy at the central TB dispensary. Once the patient tests smear-negative, (s)he is referred to a TB cabinet or polyclinic closest to their home for 2–5 months of outpatient continuation therapy. Directly observed therapy is the standard of care, requiring direct contact between patients and providers to administer the TB medication. According to the National TB Program (NTP), adherence to therapy is insufficiently controlled, with a 7.6% national default rate in 2010 [15].

In 2010, the Ukraine Red Cross Society (URCS), funded by the United States Agency for International Development (USAID), piloted a community-based social support program designed to improve TB treatment adherence during outpatient continuation therapy. URCS provided DOTS to a limited number of patients in their homes. Additionally, incentive food packages, psychological and career counseling, and/or vouchers for transportation or other necessities were provided periodically based on client needs. TB physicians managing patients’ continuation treatment at the outpatient facility made case-by-case referrals to URCS based on identification of the patient as high risk for defaulting on treatment according to established criteria for program inclusion. In 2011, the URCS program was suspended due to insufficient funds. By 2012, the program was again active and expanded to cover 10 oblasts in eastern and southern Ukraine with reported default rates ranging from 6.1–12.7% [15].

This study measures the effect of the URCS social support program on the rate of treatment default among those at risk for defaulting during continuation therapy. Given the high rate of treatment default and the growing problem of treatment-resistant TB strains, identifying effective strategies for treatment adherence is critical.

## Methods

### Study settings and sample population

Three oblasts in Ukraine were purposively chosen for this study due to their high TB caseloads and high treatment default rates. In 2010, Dnipropetrovsk reported 1,077 TB cases and 12.4% default rate; Kharkiv reported 738 cases with 11.1% default; and Odessa reported 789 cases and 9.4% default rate [15]. The study population was composed of patients receiving TB continuation treatment in these three oblasts in 2011 and 2012. Patients were classified as high-risk for TB treatment default per program criteria covering eleven self-reported risk factors: HIV positive, alcoholism, injection drug use (IDU), a contact to a TB case, a co-morbidity, homeless, unemployed, a health care worker, a migrant, a refugee or immigrant, an ex-prisoner, and room to record other risk factors that a provider might deem noteworthy. Low-risk patients did not report these risk factors with the exception of unemployment. Facility staff revealed that TB patients routinely report being unemployed. This is to avoid stigma in the workplace, as workplace TB screening is routinely undertaken after a case is diagnosed. Consequently, we considered a patient as low risk during sample selection if the only reported risk factor was unemployment. During data cleaning, minimal corrections were made for misclassification of risk status at time of data entry. All study patients completed TB intensive treatment, initiated TB continuation treatment, and had a TB treatment outcome recorded in their medical record.

Five patient cohorts were sampled: high-risk (HR) patients enrolled in the URCS social support program January 1 –May 31, 2012 (henceforth 2012 HR-Intervention); high-risk patients not enrolled in the social support program in January 1 –May 31, 2012 (henceforth 2012 HR-Comparison); low-risk (LR) patients not enrolled in the social support program in January 1 –May 31, 2012 (henceforth 2012 LR-Comparison); high-risk patients not enrolled in the social support program in January 1 –May 31, 2011 (henceforth 2011 HR-Comparison); and low-risk patients not enrolled in the social support program in January 1 –May 31, 2011 (henceforth 2011 LR-Comparison). A cohort of 2012 HR-Intervention patients (n = 409) was randomly sampled from a complete listing of URCS enrollees, stratified and proportionate in size to the TB patient population by oblast. These patients served as the index cases. Each TB facility where an index case was receiving continuation therapy served as the facility match point to ensure controls experienced similar service environments to the randomly selected cases. In order to obtain a group of controls not exposed to the program, four patients from these facilities’ TB registries were matched to the index patient: one 2012 HR-Comparison patient; one 2012 LR-Comparison patient; one 2011 HR-Comparison patient; and one 2011 LR-Comparison patient. The primary comparison group for this analysis was the 2012 HR-Comparison cohort; however, additional cohorts were sampled to explore selection. The second matching variable was the start date for TB continuation treatment for the index case in order to control for seasonality of TB and services. Additional matching on sex and age was done if more than one match was eligible. Data from 1630 TB patients across the five cohorts were collected. Sampling weights were generated to account for sampling proportionate to the varying size of TB caseload per oblast and non-response. During data cleaning, minimal corrections were made for misclassification of risk status at time of data entry. Data entry misclassifications included: dropping cases who received the intervention in 2012 but had no reported risk factors (n = 12); reclassifying those whose only risk factor was unemployment from high risk to low risk (n = 16); and reclassifying another 33 cases as high risk based on a risk factor reported.

A survey of 50 TB polyclinics and cabinets that provided continuation therapy to the study population was also completed to provide details on the referral and treatment practices at these facilities (see report for details [16]).

### Data collection and definitions

For each study patient, retrospective data were abstracted from TB medical records (national form TB01). The data abstracted included basic demographics, sex, age, employment status, urban or rural residence; and TB diagnosis, treatment, interruptions to intensive treatment and treatment outcomes. Standard WHO definitions were used for TB classification (e.g., first diagnosis, re-initiated treatment, treatment failure, relapse, and referral); for clinical TB (e.g., pulmonary or extra-pulmonary); for WHO diagnostic categories to indicate treatment regimens [17]; and for treatment outcomes (e.g., success, death, treatment failure, treatment interrupted, and transferred). A patient’s outcome was successful if the full course of prescribed treatment was completed or follow-up testing indicated patient was cured. Treatment default included anyone who missed treatment for more than 60 consecutive days per WHO standards. Additional data from the TB records were abstracted from form TB01-01, the risk screening form used by providers to identify a patient’s risk for defaulting on treatment. For the 2012 HR-Intervention group, data on social support services received such as home visits, food packages, clothing, transport vouchers, monetary incentives, and counseling were abstracted from the URCS records and merged with the patient TB record.

### Data analysis

Descriptive statistics were generated to compare demographics, TB disease characteristics, and reported risk factors for treatment default across the five cohorts. Logistic regression models with average marginal effects (AME) were estimated to test the study questions. All analyses used data weighted for sample selection; reported standard errors are clustered at the facility level.

To validate risk factors predictive of treatment default, the social support program criteria risk factors were regressed on treatment default among patients receiving continuation therapy. Dichotomous variables for seven individual risk factors (HIV positive, alcoholic, IDU, contact to a case, co-morbidity, homeless, and unemployed) were included. Very few patients reported being a health care worker, migrant, refugee, or ex-prisoner; hence, these were combined with the unspecified risk factor as “other”. A dichotomous variable indicating the presence of more than one risk factor was also added. All risk factors were run simultaneously first (Model A). Next, we controlled for basic demographics and four dichotomous disease and treatment characteristics due to their hypothesized role in TB treatment adherence and outcome: first time TB diagnosis, pulmonary TB, WHO Category I and more than 2 interruptions in care during intensive therapy (Model B). To identify the salient risk factors for default in the absence of an intervention, this analysis was restricted to data from 2011 when the URCS program was not operating.

To evaluate the impact of the social support program on treatment default, the second regression analysis was limited to the 2012 HR-Intervention and the 2012 HR-Comparison cohorts. Prior to estimating impact, balance between the intervention and comparison groups was examined. The final model estimated the impact of the social support program on treatment default, controlling for risk, disease status, and demographics.

Analyses were produced using Stata SE version 13 (College Station, TX). This study was approved by the Office of Human Research Ethics at the University of North Carolina at Chapel Hill and the ethical review board of the F.H. Yanovskyi Institute of Phthisiology and Pulmonology, Academy of Medical Sciences of Ukraine. Both review committees waived the requirement for informed patient consent. Data collection was performed by the IFAK Institut in Ukraine. Data collectors had access to patient names in order to track patients from registry entries to patient records; however, names were not recorded on data collection tools nor reported to the researchers.

## Results

### Study population

The final dataset included 1,618 records from TB patients across the five cohorts: 2011 LR-Comparison (n = 308), 2011 HR-Comparison (n = 340), 2012 LR-Comparison (n = 262), 2012 HR-Comparison (n = 311), and 2012 HR-Intervention (n = 397). The study populations shared similar demographic profiles across risk cohorts and years (Table 1). Approximately two-thirds of the patients were male in every risk group, just over three-quarters were under fifty years of age, and a large majority lived in urban areas. Over half of all patient cohorts were unemployed, ranging from 55–72%. Half of the study population received TB continuation treatment in Dnipropetrovsk (50.0%), with the remainder evenly divided between Kharkiv (25.4%) and Odessa (24.6%).

**Table 1:** Background characteristics of TB patients by year and risk cohort. Ukraine, 2011 and 2012.

	CY 2011				CY 2012						Total Patients	
	Low Risk Comparison		High Risk Comparison		Low Risk Comparison		High Risk Comparison		High Risk Intervention			
Background characteristics	*Number*	(Percent)	*Number*	(Percent)	*Number*	(Percent)	*Number*	(Percent)	*Number*	(Percent)	*Number*	(Percent)
**Sex**												
Female	121	(39.2)	114	(33.5)	87	(33.1)	102	(32.6)	154	(38.8)	577	(35.6)
Male	187	(60.8)	226	(66.5)	175	(66.9)	210	(67.4)	243	(61.2)	1041	(64.4)
**Age**												
18–29 years	92	(29.9)	52	(15.3)	73	(27.9)	37	(11.8)	96	(24.1)	349	(21.6)
30–39 years	65	(21.1)	109	(32.1)	79	(30.2)	97	(31.2)	103	(26.0)	453	(28.0)
40–49 years	55	(17.8)	95	(28.0)	52	(19.8)	87	(27.9)	97	(24.5)	386	(23.8)
50–59 years	58	(18.9)	61	(17.9)	43	(16.4)	56	(17.9)	59	(14.9)	277	(17.1)
60 and older	37	(12.0)	23	(6.7)	15	(5.7)	35	(11.2)	42	(10.6)	152	(9.4)
Missing	1	(0.3)	0	(0.0)	0	(0.0)	0	(0.0)	0	(0.0)	1	(0.1)
**Employment**												
Employed	90	(29.2)	45	(13.2)	87	(33.2)	65	(20.8)	53	(13.3)	340	(21.0)
Unemployed	168	(54.6)	243	(71.6)	146	(55.7)	190	(61.0)	276	(69.5)	1023	(63.2)
Retired/Disabled	31	(10.0)	48	(14.0)	12	(4.6)	51	(16.3)	63	(15.9)	204	(12.6)
Other	15	(4.9)	3	(0.9)	15	(5.7)	4	(1.3)	5	(1.3)	42	(2.6)
Missing	4	(1.3)	1	(0.3)	2	(0.8)	2	(0.6)	0	(0.0)	9	(0.6)
**Residence**												
Rural	46	(15.0)	69	(20.4)	47	(18.0)	45	(14.5)	55	(13.9)	263	(16.2)
Urban	262	(85.0)	270	(79.3)	215	(82.0)	266	(85.5)	342	(86.1)	1354	(83.7)
Missing	0	(0.0)	1	(0.3)	0	(0.0)	0	(0.0)	0	(0.0)	1	(0.1)
**Oblast**												
Dnipropetrovsk	160	(52.0)	147	(43.2)	136	(51.9)	154	(49.4)	213	(53.7)	810	(50.0)
Kharkiv	74	(24.0)	112	(33.0)	64	(24.3)	69	(22.1)	92	(23.2)	410	(25.4)
Odessa	74	(24.0)	81	(23.8)	62	(23.8)	89	(28.5)	92	(23.1)	398	(24.6)
**Total Patients**	308	(100.0)	340	(100.0)	262	(100.0)	311	(100.0)	397	(100.0)	1618	(100.0)

Among the three HR cohorts, unemployment was the most common reported risk factor (48–62 percent), followed by alcoholism (34–44 percent), co-morbidities (33–36 percent) and being HIV-positive (21–47 percent) (Table 2). A majority (63–72 percent) reported between 2 and 3 factors putting them at risk for treatment default, while 3–7 percent reported four or more. Notably, the proportion of HR patients who reported injection drug use in their medical records was small, ranging from 5–12 percent. In discussions with facility staff, it was noted that information on IDU status and treatment is not routinely recorded in the TB charts nor shared across cabinets due to concerns of confidentiality. As expected over half of the LR patients reported no risk factors for treatment default, while the remaining reported unemployment.

**Table 2:** TB Patient risk profiles by year and risk cohort. Ukraine, 2011 and 2012.

	CY 2011				CY 2012						Total Patients	
	Low Risk Comparison		High Risk Comparison		Low Risk Comparison		High Risk Comparison		High Risk Intervention			
Risk Profile	*Number*	(Percent)	*Number*	(Percent)	*Number*	(Percent)	*Number*	(Percent)	*Number*	(Percent)	*Number*	(Percent)
Risk Factor*												
HIV-positive	0	(0.0)	114	(33.5)	0	(0.0)	133	(42.6)	83	(20.8)	329	(20.3)
Alcoholic	0	(0.0)	139	(41.0)	0	(0.0)	105	(33.7)	174	(43.8)	418	(25.9)
Injection Drug User	0	(0.0)	40	(11.9)	0	(0.0)	24	(7.8)	19	(4.8)	84	(5.2)
Contact to Case	0	(0.0)	26	(7.6)	0	(0.0)	21	(6.7)	23	(5.8)	70	(4.3)
Co-morbidity	0	(0.0)	113	(33.2)	0	(0.0)	113	(36.1)	143	(36.1)	369	(22.8)
Homeless	0	(0.0)	11	(3.2)	0	(0.0)	18	(5.7)	7	(1.8)	36	(2.2)
Unemployed	120	(38.9)	199	(58.6)	119	(45.6)	151	(48.3)	245	(61.8)	834	(51.6)
Health Care Worker	0	(0.0)	7	(2.1)	0	(0.0)	10	(3.2)	6	(1.5)	23	(1.4)
Migrant	0	(0.0)	0	(0.0)	0	(0.0)	2	(0.6)	4	(1.0)	6	(0.4)
Refugee/Immigrant	0	(0.0)	2	(0.6)	0	(0.0)	0	(0.0)	2	(0.5)	4	(0.2)
Ex-Prisoner	0	(0.0)	16	(4.7)	0	(0.0)	16	(5.1)	7	(1.8)	39	(2.4)
Other	0	(0.0)	34	(10.1)	0	(0.0)	32	(10.4)	78	(19.7)	145	(9.0)
**Number of Risk Factors**												
No risk factors	188	(61.1)	0	(0.0)	143	(54.4)	0	(0.0)	0	(0.0)	330	(20.4)
1	120	(38.9)	85	(24.9)	119	(45.6)	94	(30.3)	102	(25.6)	521	(32.2)
2–3	0	(0.0)	244	(71.8)	0	(0.0)	197	(63.3)	277	(69.8)	718	(44.4)
4 or more	0	(0.0)	11	(3.3)	0	(0.0)	20	(6.4)	18	(4.6)	49	(3.0)
**Total Patients**	308	(100.0)	340	(100.0)	262	(100.0)	311	(100.0)	397	(100.0)	1618	(100.0)

*Multiple responses possible, may not sum to 100%

Overall, 81.1 percent of the TB patients were undergoing treatment for a first diagnosis, although among the HR cohorts, a higher percentage re-initiated treatment after earlier failure or relapse compared to the LR cohorts (7–12 percent versus 3–5 percent) (Table 3). Ninety-three percent of all cases were pulmonary TB, a majority was classified as WHO Category I (63.8 percent), and 81.3 percent reported only one or fewer interruptions in intensive treatment.

**Table 3:** TB patient's disease status and treatment outcome by risk cohort and year. Ukraine, 2011 and 2012.

	CY 2011				CY 2012							Total Patients
	Low Risk Comparison		High Risk Comparison		Low Risk Comparison		High Risk Comparison		High Risk Intervention			
Disease Status	*Number*	(Percent)	*Number*	(Percent)	*Number*	(Percent)	*Number*	(Percent)	*Number*	(Percent)	*Number*	(Percent)
**TB Classification**												
First Diagnosis	274	(89.0)	273	(80.3)	224	(85.5)	242	(77.6)	300	(75.5)	1312	(81.1)
Re-Initiated	15	(4.9)	37	(10.9)	9	(3.5)	22	(7.1)	46	(11.6)	129	(8.0)
Relapse	19	(6.1)	30	(8.8)	27	(10.3)	47	(15.0)	47	(11.8)	170	(10.5)
Referral	0	(0.0)	0	(0.0)	2	(0.8)	1	(0.3)	4	(1.0)	7	(0.4)
**TB Clinical Form**												
Pulmonary	289	(93.8)	303	(89.1)	247	(94.3)	292	(93.9)	374	(94.2)	1505	(93.0)
Extra-Pulmonary	19	(6.2)	36	(10.6)	15	(5.7)	19	(6.1)	23	(5.8)	112	(6.9)
**WHO Treatment Category**												
Category I	191	(62.0)	224	(65.9)	163	(62.1)	214	(68.6)	242	(60.8)	1032	(63.8)
Category II	34	(11.0)	69	(20.3)	43	(16.5)	70	(22.4)	89	(22.5)	305	(18.8)
Category III	83	(27.0)	46	(13.6)	56	(21.5)	28	(9.0)	62	(15.7)	276	(17.0)
Other	0	(0.0)	0	(0.0)	0	(0.0)	0	(0.0)	3	(0.8)	3	(0.2)
Missing	0	(0.0)	1	(0.3)	0	(0.0)	0	(0.0)	1	(0.3)	2	(0.1)
**Intensive Treatment Interruptions**												
None	224	(72.6)	207	(60.8)	194	(74.0)	228	(73.3)	269	(67.8)	1123	(69.4)
1 interruption	30	(9.7)	57	(16.8)	24	(9.2)	25	(8.0)	56	(14.1)	192	(11.9)
2–3 interruptions	26	(8.5)	57	(16.8)	32	(12.2)	37	(11.9)	48	(12.1)	200	(12.4)
≥ 4 interruptions	14	(4.5)	10	(2.9)	5	(1.9)	17	(5.5)	20	(5.0)	66	(4.1)
Missing	14	(4.6)	9	(2.7)	7	(2.7)	4	(1.3)	4	(1.0)	38	(2.4)
**TB Treatment Outcome**												
Success	279	(90.6)	253	(74.3)	228	(87.0)	217	(69.8)	351	(88.4)	1327	(82.0)
Died	0	(0.0)	14	(4.1)	4	(1.5)	25	(8.1)	8	(2.0)	51	(3.2)
Treatment failed	13	(4.2)	25	(7.4)	17	(6.5)	33	(10.6)	31	(7.8)	119	(7.4)
Treatment default	13	(4.2)	45	(13.3)	12	(4.6)	33	(10.6)	5	(1.3)	108	(6.7)
Transferred	3	(1.0)	3	(0.9)	1	(0.4)	3	(1.0)	2	(0.5)	12	(0.7)
**Total Patients**	308	(100.0)	340	(100.0)	262	(100.0)	311	(100.0)	397	(100.0)	1618	(100.0)

TB treatment outcomes in 2011 were significantly different between the LR and HR cohorts on treatment adherence. Treatment default among the 2011 LR-Comparison cohort was 4.2 percent compared to 13.3 percent in the 2011 HR-Comparison cohort (p<0.000); while 90.6 percent of the LR-Comparison cohort reported treatment success compared to only 74.3 percent of the HR cohort (p<0.000) (Table 3). Similar differences were measured in 2012 when comparing the LR-Comparison and HR-Comparison on default, 4.6 percent and 10.6 percent (p<0.006), and success, 87.0 percent and 69.8 percent (p<0.000) respectively. The 2012 HR-Intervention cohort fared better than the 2012 HR-Comparison on default (1.3 and 10.6 respectively, p<0.000) and on success (88.4 and 69.8 respectively, p<0.000). However, comparisons between the 2012 LR-Comparison and the 2012 HR-Intervention on default (4.6 and 1.3 percent respectively) and success (87.0 and 88.4 percent respectively) found no statistical differences. Lastly, statistical differences were found between the 2011 HR-Comparison and the 2012 HR-Intervention cohorts for both treatment default and treatment success (p<0.000); while no statistical differences were found between the 2011 HR-Comparison and 2012 HR-Comparison groups. These comparisons across cohorts highlight that the difference in outcomes between the 2012 HR and LR comparison cohorts is similar to the differences between the 2011 HR and LR cohorts. This supports the impact identification strategy employed in our evaluation of the program.

### Predicting treatment default

The URCS social support program was designed to target those at highest risk of treatment default and provide support to improve treatment adherence. The official eligibility criteria for program support cover eleven risk factors. Among patients from 2011, only those who reported being an alcoholic (p = 0.002) or an IDU (p = 0.043) were more likely to default on TB continuation treatment, while a patient reporting a co-morbidity was less likely to default (p = 0.028) (Table 4). Additionally, those patients enrolled in continuation care who had two or more interruptions recorded during intensive treatment were more likely to default during outpatient treatment (p = 0.028). Estimated marginal effects predict that an individual’s probability of default increased by 0.08 (p = 0.017) if an alcohol abuser and by 0.05 (p = 0.043) if prior treatment interruptions were noted; yet one’s probability of default decreased by 0.06 (p = 0.043) if reporting a co-morbidity.

**Table 4:** Risk factors predictive of TB treatment default. Ukraine, 2011.

	Model A						Model B					
	Coeff.	SE	p-value	AME	SE	p-value	Coeff.	SE	p-value	AME	SE	p-value
**HIV-positive**	-0.299	(0.519)	(0.567)	-0.020	(0.033)	(0.536)	-0.597	(0.480)	(0.218)	-0.037	(0.026)	(0.152)
**Alcoholic**	1.232**	(0.370)	(0.001)	0.107*	(0.047)	(0.027)	1.002**	(0.317)	(0.002)	0.080*	(0.033)	(0.017)
**Injection Drug User**	0.701	(0.409)	(0.091)	0.061	(0.040)	(0.129)	0.800*	(0.388)	(0.043)	0.066	(0.040)	(0.100)
**Contact to Case**	-0.626	(0.776)	(0.422)	-0.038	(0.043)	(0.389)	-0.391	(0.786)	(0.621)	-0.024	(0.046)	(0.600)
**Co-morbidity**	-1.046*	(0.505)	(0.042)	-0.062	(0.035)	(0.083)	-0.954*	(0.425)	(0.028)	-0.055*	(0.027)	(0.043)
**Homeless**	0.245	(0.992)	(0.805)	0.019	(0.083)	(0.819)	0.556	(1.008)	(0.583)	0.044	(0.093)	(0.636)
**Unemployed**	0.341	(0.465)	(0.467)	0.024	(0.030)	(0.418)	0.351	(0.456)	(0.445)	0.023	(0.029)	(0.418)
**Other**^**1**^	-0.714	(0.653)	(0.278)	-0.043	(0.038)	(0.258)	-0.644	(0.747)	(0.391)	-0.038	(0.041)	(0.362)
**> 1 Risk Factor**	1.098	(0.573)	(0.059)	0.082	(0.059)	(0.170)	0.992	(0.556)	(0.079)	0.070	(0.050)	(0.170)
**First TB Diagnosis**							0.061	(0.369)	(0.870)	0.004	(0.025)	(0.869)
**Pulmonary TB**							-0.870	(0.458)	(0.062)	-0.071	(0.048)	(0.140)
**WHO Category I**							0.149	(0.343)	(0.664)	0.010	(0.023)	(0.658)
**≥ 2 Interruptions**							0.691*	(0.309)	(0.028)	0.054*	(0.026)	(0.043)
**Male**							-0.588	(0.321)	(0.072)	-0.043	(0.027)	(0.115)
**Age Group**												
18–29 years (ref.)												
30–39 years							0.290	(0.696)	(0.678)	0.018	(0.039)	(0.649)
40–49 years							0.492	(0.693)	(0.480)	0.032	(0.039)	(0.413)
50–59 years							0.617	(0.454)	(0.179)	0.042	(0.028)	(0.142)
60 and older							-0.560	(1.141)	(0.625)	-0.026	(0.051)	(0.612)
**Urban Residence**							-0.599	(0.355)	(0.096)	-0.046	(0.030)	(0.126)
**Oblast**												
Dnipropetrovsk (ref.)												
Kharkiv							0.247	(0.456)	(0.590)	0.018	(0.036)	(0.617)
Odessa							-0.342	(0.626)	(0.586)	-0.021	(0.035)	(0.551)
Constant	-3.324***	(0.332)	(0.000)				-2.289***	(0.586)	0.000			
N	648			648			622			622		

^1^ Other includes health care worker, migrant, refugee, ex-prisoner and other unspecified.

* p<0.05

**p<0.01

*** p<0.001

### Evaluating program impact

Primary impact analyses were limited to the 2012 HR-Intervention and the 2012 HR-Comparison cohorts. Mean differences between the two groups were tested for 18 variables; seven (38 percent) were unbalanced at standard statistical levels (p<0.05). Looking at the intervention group, a higher proportion of patients were alcoholics or unemployed, and were 18–29 years of age, while the comparison group had a higher proportion of persons with HIV, homeless, undergoing WHO treatment category 1, and who were male. All risk factors, disease characteristics, patient demographics and treatment oblast were controlled for in the final impact model. Measuring impact, results indicate that the HR patients receiving the social support program decreased their probability of treatment default by 0.101 (p<0.000) compared to the comparison cohort (Table 5).

**Table 5:** Impact of social support program on treatment default among high-risk TB patients, Ukraine, 2012.

	2012 Cohort					
	Coefficient	(SE)	(p-value)	AME	(SE)	(p-value)
**Social Support Intervention**	-2.506***	(0.458)	(0.000)	-0.101***	(0.025)	(0.000)
**HIV-positive**	0.041	(0.567)	(0.942)	0.002	(0.025)	(0.942)
**Alcoholic**	0.958	(0.530)	(0.075)	0.045	(0.024)	(0.069)
**Injection Drug User**	0.978	(0.601)	(0.108)	0.057	(0.046)	(0.218)
**Contact to Case**	-1.284	(1.152)	(0.269)	-0.038	(0.021)	(0.068)
**Co-morbidity**	-0.292	(0.607)	(0.632)	-0.012	(0.025)	(0.629)
**Homeless**	1.110	(0.597)	(0.067)	0.068	(0.046)	(0.145)
**Unemployed**	0.503	(0.414)	(0.229)	0.022	(0.017)	(0.219)
**Other**^**1**^	-0.273	(0.749)	(0.717)	-0.011	(0.029)	(0.696)
**> 1 Risk Factor**	0.181	(0.746)	(0.809)	0.008	(0.031)	(0.804)
**First TB Diagnosis**	-0.160	(0.917)	(0.862)	-0.007	(0.043)	(0.865)
**Pulmonary TB**	-0.084	(0.783)	(0.915)	-0.004	(0.037)	(0.917)
**WHO Category I**	0.867	(0.891)	(0.334)	0.035	(0.032)	(0.283)
**≥ 2 Interruptions**	0.514	(0.522)	(0.328)	0.025	(0.027)	(0.352)
**Male**	0.579	(0.451)	(0.204)	0.023	(0.017)	(0.176)
**Age Group**						
18–29 years (ref.)						
30–39 years	-0.194	(0.558)	(0.729)	-0.012	(0.034)	(0.733)
40–49 years	-0.862	(0.605)	(0.159)	-0.042	(0.030)	(0.165)
50–59 years	-0.752	(0.588)	(0.205)	-0.038	(0.030)	(0.213)
60 and older	-1.785	(1.151)	(0.125)	-0.065	(0.033)	(0.053)
**Urban Residence**	0.325	(0.641)	(0.614)	0.013	(0.024)	(0.582)
**Oblast**						
Dnipropetrovsk (ref.)						
Kharkiv	0.330	(0.529)	(0.535)	0.015	(0.025)	(0.547)
Odessa	0.031	(0.701)	(0.964)	0.001	(0.030)	(0.965)
Constant	-3.673*	(1.575)	(0.023)			
N	706			706		

^1^ Other includes health care worker, migrant, refugee, ex-prisoner and other unspecified.

*p<0.05

**p<0.01

***p<0.001

A second analysis, comparing outcomes for the 2012 HR-Intervention cohort to the 2011 HR-Comparison cohort, produced similar results. Five of 18 variables (28 percent) were not balanced between the cohorts. Controlling for all variables, the 2012 HR patients receiving the social support intervention were significantly less likely to default (p<0.000) compared to the 2011 HR-Comparison cohort, and the probability of default decreased by 0.120 (p<0.000) (data not shown). However, alcoholism remained a significant risk factor with the probability of default 0.069 (p<0.014) higher among alcoholics compared to non-alcoholics. Additionally, the probability of default among those with more than two treatment interruptions during intensive care was higher at 0.047 (p = 0.017).

## Discussion

In 2012, TB patients receiving social support provided by URCS in Ukraine reduced their probability of defaulting on continuation treatment by 10 percentage points compared to high-risk patients who did not receive social support in 2012 or 2011. Treatment success rates for the high-risk patients receiving social support were comparable to the low-risk cohorts and significantly improved over the high-risk comparison cohorts. This result was found despite the heterogeneity of the patient population and the services provided.

Although treatment oblast was not predictive of success or default in 2012, routine implementation of DOTS and social support varied by study oblast. According to reported practices in 2014, 89 percent of surveyed facilities in Dnipropetrovsk provided facility-based DOTS and a majority of these facilities required daily DOTS visits (83 percent) [16]. Among the sites offering home-based DOTS, half provided weekly or bi-weekly visits. In contrast, 88 percent of the facilities in Odessa provided home-based DOTS and the majority of home visits were daily. This increase in home-based services may reflect a growing recognition in Odessa that facility DOTS is insufficient to assure compliance. Variation across oblasts may reflect patient population needs or facility capacity. Further investigation into best practices for DOTS and social support in Ukraine is warranted.

URCS was the only provider of social support in Kharkiv and Odessa in 2012 and the primary provider in Dnipropetrovsk. In 2011, only 23 percent of the facilities referred patients for social support, increasing to 94 percent by 2012. In all oblasts the primary point of referral was the city or raion TB physician. This is in keeping with URCS’ policy to only provide social support to smear-negative patients who successfully completed intensive TB treatment and initiated continuation treatment. This focus on continuation patients ignored patients who defaulted during intensive treatment, which could be substantial. In Russia, Jakubowiak et al. found that 44 percent of TB treatment defaulters exited treatment during the intensive regimen [18]. In Moldova, the highest default rates were recorded during the first month of inpatient intensive treatment [10]. Our data did not include patients who defaulted during inpatient treatment, however in 2011, patients with more than two treatment interruptions during inpatient care increased their probability of defaulting during outpatient care by 5 percentage points. This is similar to findings by Jakubowiak in Russia and Santha in India, where gaps in intensive treatment were associated with future treatment default [18,19]. Prioritization for support services may benefit those who had difficulty during the intensive phase.

Alcoholism was the one risk criteria predictive of defaulting among the 2011 cohorts, increasing the probability of default by 5 percentage points. Neither positive HIV status nor reported injection drug use were statistically associated with higher default rates. Under-reporting of these two risk factors may be one explanation for their lack of significance. In Ukraine, sharing of confidential patient information between service delivery clinics is limited. The risk factor information documented on a patient’s TB form is all self-reported and possibly under-reported due to fear of stigmatization. For example, a patient seeking HIV-related services may not report their status to the TB physician. Unless an infectious disease specialist is overseeing services for both TB and HIV patients, this case of co-infection may go undetected by the individual clinics, despite best practices of routine HIV screening among TB patients. According to the facility survey, only 32% of facilities providing DOTS also provided ART for persons living with HIV. For IDUs the challenge may be two-fold. First, the availability of drug-substitution therapy for IDUs in Ukraine is scarce; only 16% of the outpatient TB facilities reported offering this service. Without adequate substitution therapy, many IDUs may drop out of service during the intensive, inpatient TB treatment phase, excluding them from our sample. Second, the stigma for drug addiction may discourage IDUs from revealing this risk to their TB physician. In either scenario, the risk of default among IDUs may not be adequately reflected in our data.

Patients with reported co-morbidities reduced their probability of defaulting by almost 6 percentage points in 2011, possibly due to additional support received from providers caring for the co-morbidities. For all other risk factors, no statistical associations were found with default. Whether this is due to the small numbers of patients with these other risks or because these factors do not increase one’s risk of default is undetermined in this study. Interestingly, almost 20% of the high-risk cohort receiving the intervention had an undetermined or “other” risk factor recorded. Provider interviews suggested that compliant patients in our study sites may have been referred to URCS as a reward for their adherence. If widespread, this preferential referral of adherent patients could create selection bias, affecting results. This is one of the limitations of retrospective data analysis, it is difficult to measure the fidelity of program implementation retrospectively. However, if there was widespread selective referral one would expect the 2012 HR-Comparison group to have reported a higher default rate than the 2011 HR-Comparison group. The comparability of the default rates among these two cohorts suggests that very little selection bias exists.

In an era of declining health resources and increasing drug-resistant TB, refining and standardizing the referral criteria for additional social support may reduce the national default rate, but not without a cost. This study shows that social support is effective in reducing default rates but whether or not that means it should or can be scaled-up depends on the cost of wider implementation and the cost relative to other potential interventions that might also reduce default rates.

## Conclusions

This study demonstrates the positive impact of providing social support to those at-risk for treatment default. Targeting services to those who will most benefit is critical to reduce continuing TB transmission. Further research is recommended to differentiate the costs and benefits from home-based DOTS versus additional services offered through social support programs. Prospective cohort studies could refine targeting of programming, evaluate social support program fidelity, identify which populations respond best to select services, and what barriers might still exist to achieving better adherence. With that information, tailoring programs to most effectively reach and serve clients in a patient-centered approach may reap substantial rewards for Ukraine. Prioritizing support services for clients who struggle with alcohol or drug addictions or struggle with adherence to intensive inpatient treatment regimens, may improve treatment success. Identifying approaches to assure intensive treatment completion and flagging those upon completion for additional follow-up during continuation treatment, has the potential to further reduce program defaults and improve outcomes.

## References

[1] World Health Organization. Global tuberculosis report 2016. Geneva: 2016. Available from: http://www.who.int/tb/publications/global_report/en/

[2] Obermeyer Z, Abbott-Klafter J, Murray CJ. Has the DOTS strategy improved case finding or treatment success? An empirical assessment. PLoS One. 2008 3 5;3(3):e1721 10.1371/journal.pone.0001721 18320042 PMC2253827

[3] Akachi Y, Zumla A, Atun R. Investing in improved performance of national tuberculosis programs reduces the tuberculosis burden: analysis of 22 high-burden countries, 2002–2009. Journal of Infectious Diseases. 2012 3 28:jis189. 10.1093/infdis/jis189 22457297

[4] Marx FM, Dunbar R, Enarson DA, Beyers N. The rate of sputum smear-positive tuberculosis after treatment default in a high-burden setting: a retrospective cohort study. PLoS One. 2012 9 25;7(9):e45724 10.1371/journal.pone.0045724 23049846 PMC3458061

[5] Dubrovina I, Miskinis K, Lyepshina S, Yann Y, Hoffmann H, Zaleskis R, et al Drug-resistant tuberculosis and HIV in Ukraine: a threatening convergence of two epidemics? The International Journal of Tuberculosis and Lung Disease. 2008 7 1;12(7):756–62. 18544200

[6] Kruk ME, Schwalbe NR, Aguiar CA. Timing of default from tuberculosis treatment: a systematic review. Tropical Medicine & International Health. 2008 5 1;13(5):703–12. 18266783 10.1111/j.1365-3156.2008.02042.x

[7] Bustamante-Montes LP, Escobar-Mesa A, Borja-Aburto VH, Gomez-Munoz A, Becerra-Posada F. Predictors of death from pulmonary tuberculosis: the case of Veracruz, Mexico. The International Journal of Tuberculosis and Lung Disease. 2000 3 1;4(3):208–15. 10751065

[8] Caylà JA, Rodrigo T, Ruiz-Manzano J, Caminero JA, Vidal R, García JM, et al Tuberculosis treatment adherence and fatality in Spain. Respiratory Research. 2009 12 1;10(1):121. 19951437 10.1186/1465-9921-10-121 PMC2794858

[9] Jakubowiak WM, Bogorodskaya EM, Borisov ES, Danilova DI, Kourbatova EK. Risk factors associated with default among new pulmonary TB patients and social support in six Russian regions. The International Journal of Tuberculosis and Lung Disease. 2007 1 1;11(1):46–53. 17217129

[10] Jenkins HE, Ciobanu A, Plesca V, Crudu V, Galusca I, Soltan V, et al Risk factors and timing of default from treatment for non-multidrug-resistant tuberculosis in Moldova. The International Journal of Tuberculosis and Lung Disease. 2013 3 1;17(3):373–80. 10.5588/ijtld.12.0464 23407226 PMC3710709

[11] Kliiman K, Altraja A. Predictors and mortality associated with treatment default in pulmonary tuberculosis. The International Journal of Tuberculosis and Lung Disease. 2010 4 1;14(4):454–63. 20202304

[12] Toczek A, Cox H, Du Cros P, Cooke G, Ford N. Strategies for reducing treatment default in drug-resistant tuberculosis: systematic review and meta-analysis [Review article]. The International Journal of Tuberculosis and Lung Disease. 2013 3 1;17(3):299–307. 10.5588/ijtld.12.0537 23211716

[13] Wandwalo E, Kapalata N, Egwaga S, Morkve O. Effectiveness of community-based directly observed treatment for tuberculosis in an urban setting in Tanzania: a randomised controlled trial. The International Journal of Tuberculosis and Lung Disease. 2004 10 1;8(10):1248–54. 15527158

[14] Jakubowiak WM, Bogorodskaya EM, Borisov SE, Danilova ID, Lomakina OB, Kourbatova EV. Impact of socio-psychological factors on treatment adherence of TB patients in Russia. Tuberculosis. 2008 9 30;88(5):495–502. 10.1016/j.tube.2008.03.004 18501675

[15] Ukraine Ministry of Health. National Tuberculosis Program data, 2010. Kyiv: 2010.

[16] Strengthening Tuberculosis Control in Ukraine: Impact Evaluation Baseline Survey, Ukraine 2014. MEASURE Evaluation: 2015. Available from: https://www.measureevaluation.org/resources/publications/tr-15-116

[17] World Health Organization (2003). Treatment of tuberculosis: guidelines for national programmes, third edition Geneva: WHO.

[18] Jakubowiak W, Bogorodskaya E, Borisov S, Danilova I, Kourbatova E. Treatment interruptions and duration associated with default among new patients with tuberculosis in six regions of Russia. International Journal of Infectious Diseases. 2009 5 31;13(3):362–8. 10.1016/j.ijid.2008.07.015 19008141

[19] Santha T, Garg R, Frieden T, Chandrasekaran V, Subramani R, Gopi P, et al Risk factors associated with default, failure and death among tuberculosis patients treated in a DOTS programme in Tiruvallur District, South India, 2000. The International Journal of Tuberculosis and Lung Disease. 2002 9 1;6(9):780–8. 12234133

